# Dysregulation of intracellular redox homeostasis by the SARS-CoV-2 ORF6 protein

**DOI:** 10.1186/s12985-023-02208-7

**Published:** 2023-10-18

**Authors:** Marta De Angelis, Gabriele Anichini, Anna Teresa Palamara, Lucia Nencioni, Gianni Gori Savellini

**Affiliations:** 1 Department of Public Health and Infectious Diseases, Sapienza University, Rome, Italy; 2 Laboratory of Virology, Department of Molecular Medicine, Sapienza University, Rome, Italy; 3 Department of Medical Biotechnologies, University of Siena, Siena, Italy; 4 Department of Infectious Diseases, Istituto Superiore Di Sanità, Rome, Italy

**Keywords:** Oxidative stress, SARS-CoV-2, NRF2, Antagonistic proteins, ORF6

## Abstract

SARS-CoV-2 has evolved several strategies to overcome host cell defenses by inducing cell injury to favour its replication. Many viruses have been reported to modulate the intracellular redox balance, affecting the Nuclear factor erythroid 2-Related Factor 2 (NRF2) signaling pathway. Although antioxidant modulation by SARS-CoV-2 infection has already been described, the viral factors involved in modulating the NRF2 pathway are still elusive. Given the antagonistic activity of ORF6 on several cellular pathways, we investigated the role of the viral protein towards NRF2-mediated antioxidant response. The ectopic expression of the wt-ORF6 protein negatively impacts redox cell homeostasis, leading to an increase in ROS production, along with a decrease in NRF2 protein and its downstream controlled genes. Moreover, when investigating the Δ61 mutant, previously described as an inactive nucleopore proteins binding mutant, we prove that the oxidative stress induced by ORF6 is substantially related to its C-terminal domain, speculating that ORF6 mechanism of action is associated with the inhibition of nuclear mRNA export processes. In addition, activation by phosphorylation of the serine residue at position 40 of NRF2 is increased in the cytoplasm of wt-ORF6-expressing cells, supporting the presence of an altered redox state, although NRF2 nuclear translocation is hindered by the viral protein to fully antagonize the cell response. Furthermore, wt-ORF6 leads to phosphorylation of a stress-activated serine/threonine protein kinase, p38 MAPK, suggesting a role of the viral protein in regulating p38 activation. These findings strengthen the important role of oxidative stress in the pathogenesis of SARS-CoV-2 and identify ORF6 as an important viral accessory protein hypothetically involved in modulating the antioxidant response during viral infection.

## Background

Coronaviruses (CoVs) are single-stranded, positive-sense RNA viruses with a large genome comprising the open reading frames (ORFs) 1a and 1b. These ORFs encode two polyproteins that are proteolytically cleaved into 16 non-structural proteins (Nsp1–16) which play crucial roles in the CoV life cycle [1–3]. In addition to structural and Nsp proteins, the CoV genomes also contain information for accessory proteins, encoded by ORFs located at the 3’-end, which are mainly involved in regulating the host’s response to infection [1–3]. Since the virus outbreak at the end of 2019 [4, 5], several virus variants have been reported, including the more recent BQ.1.1, BF.7, and XBB Omicron sub-lineages [6–9]. Differences in virus replication rates have been observed for these variants, suggesting the involvement of factors beyond host cell recognition [10–14]. Viruses, including SARS-CoV-2, exploit various host cell pathways to support their replication and evade host defenses, such as suppressing immune responses [15–20]. Oxidative stress is a biological process characterized by an imbalance between oxidant and antioxidant molecules within cells, leading to the overproduction of Reactive Oxygen Species (ROS) and Reactive Nitrogen Species (RNS), causing cellular damage [21–25]. Viral infections disrupt the intracellular redox microenvironment, resulting in increased ROS production [26]. Nuclear factor erythroid 2-Related Factor 2 (NRF2) is the master transcription factor responsible for the activation of antioxidant response, protecting cells from oxidative stress and injury. Several respiratory viruses, including human Respiratory Syncytial Virus (RSV) [27–29], Influenza virus (IV) [30], and Coronaviruses (CoVs) [31–36], have been reported to modulate the redox state, affecting NRF2-mediated antioxidant response [37–40]. For instance, studies on IV have shown that reduced glutathione (GSH) levels decrease during infection, leading to an imbalance in NOX4-mediated ROS production, which activates p38 and ERK Mitogen-Activated Protein Kinase (MAPK), promoting the nuclear export of viral ribonucleoprotein [41–43]. Moreover, IV infection downregulates NRF2 gene expression and its nuclear translocation [30]. Other studies have highlighted the potential impact of oxidative stress on SARS-CoV-2 infection, suggesting that the overproduction of ROS and an impaired antioxidant system play a significant role in SARS-CoV-2 pathogenesis and the severity of Acute Respiratory Distress Syndrome (ARDS) [44–46]. Studies on COVID-19 patients have shown that severe illness is associated with lower GSH levels and higher ROS production than mild disease [32, 33]. Additionally, lung biopsies from patients with severe COVID-19 exhibit downregulation of NRF2-mediated gene expression [34], and in vitro and in vivo studies have indicated that SARS-CoV-2 infection inhibits GSH and increases its oxidative form, GSSG, leading to reduced NRF2 activity [33, 35]. Although the viral components involved in these mechanisms have not been fully investigated and characterized, recent research has shown that the SARS-CoV-2 Nsp14 protein is capable of modulating the host cellular redox state by interacting with Sirtuin 1 and 5 (SIRT1-5) [47, 48]. In this context, considering the relationship between NRF2 pathway and the innate immune response [49, 50] in the host protection against respiratory virus infections, we explore the role of SARS-COV-2 ORF6 accessory protein. ORF6 has been previously associated with the control of host innate immunity and inflammation by blocking the expression of type I interferons (IFN-β) and interleukin-6 (IL-6) by hindering mRNA migration from the nucleus to the cytoplasm for translation [51]. Indeed, ORF6 is able of binding and of inhibiting nucleopore complex (NPC) proteins, affecting mRNA export from the nucleus to the cytoplasm, leading to a shutdown of cellular protein synthesis. Given the correlation between inflammation, innate immunity, and the redox state, our hypothesis was that the ORF6 protein might be involved in the modulation of cellular antioxidant response. Our results clearly demonstrate that the ORF6 protein negatively impacts cell homeostasis, leading to a downregulation of NRF2 signaling, including the downstream Glucose-6-Phosphate Dehydrogenase (G6PD) and Heme-Oxygenase-1 (HO-1) detoxifying proteins. These results are a consequence of the overproduction of ROS caused by the ectopic expression of ORF6. However, this mechanism is unlikely to be associated with transcriptional events, as NRF2 and downstream NRF2-controlled target mRNAs are induced by ORF6-mediated cell perturbation. Furthermore, NRF2 activation through phosphorylation by the cellular p38 MAPK is not impaired by the presence of ORF6, although its nuclear translocation is hindered by the viral protein. Moreover, the ORF6 C-terminus is critical for protein function in controlling the cellular antioxidant response, as supported by evidence using two protein mutants, M_58_R and Δ61. While the M_58_R residue mutation drastically reduces protein activity, the deletion of the last amino acid D61 (Δ61) completely abrogates protein function on NRF2, as well as on IFN-β and IL-6, as previously described [51, 52].

## Methods

### Cells and chemicals

Human embryonic kidney HEK-293T cells (ATCC CRL-1573) and A549 cells (ATCC CCL-185) are cultured in Dulbecco’s modified Eagle’s medium (DMEM) (EuroClone, Milan, Italy) supplemented with 100 U/mL penicillin/streptomycin (HyClone Europe, Milan, Italy) and 10% heat-inactivated foetal bovine serum (FCS) (Lonza) at 37 °C. Transfections are performed using the GeneJuice Transfection Reagent (Merck Life Sciences, Milan, Italy) following the manufacturer’s instructions. Tert-Butylhydroquinone (tBHQ), MG-132 proteasome inhibitor and N-acetyl-cysteine (NAC) are purchased from Merck Life Science.

### Plasmids

The HA-tagged SARS-CoV-2 wild-type and M_58_R ORF6 expression plasmids are kindly provided by Prof. A. García-Sastre (CEIRS program; NIAD Centers of Excellence for Influenza Research and Surveillance; Mount Sinai School of Medicine, New York), while plasmid encoding HA-tagged SARS-CoV-2 ORF6 deleted by the last amino-acid (ORF6-∆61) mutant was prepared by standard cloning procedures using a naturally occurring virus variant (GenBank accession numbers OP002141). The ∆61 ORF6 coding sequence was amplified by Reverse-Transcription (RT)-Polymerase Chain Reaction (PCR) from viral RNA purified by using the QIAamp viral RNA mini kit (Qiagen, Milan, Italy) and cloned in the pCAGGS-MCS plasmid, in frame with a C-terminal HA-tag, at the EcoRI and XhoI unique sites. Recombinant plasmids are verified by sequencing. The pARE reporter plasmids encoding Firefly luciferase downstream of the Antioxidant Responsive Elements (ARE) and the Renilla luciferase downstream of the constitutively active SV40 promoter (pSV40-RL) are purchased from Promega (Milan, Italy). The plasmid encoding FLAG-tagged NRF2 was a gift from Randall Moon (Addgene plasmid #36971).

### Luciferase reporter assay

To study the wild-type (wt)- or mutated ORF6 protein activity towards pARE, HEK-293T or A549 (2 × 10^5^/well) cells are seeded in 24-well plates, and after over-night (o/n) incubation, the cells are transfected with 0.2 μg and 0.02 μg of pARE and pRL reporter plasmids, respectively, alone or in combination with 0.1 μg of ORF6-expressing plasmids. Where indicated, cells are co-transfected with 0.05 μg of NRF2 encoding plasmid. The total transfected DNA amount was kept constant by using an empty plasmid. At 36 h post-transfections, where indicated, cells are treated with 10 μM tBHQ, 1 μM MG-132 or an equivalent volume of vehicle for an additional 12 h. Cells are collected, and luciferase activities are measured on lysates using the Dual-Luciferase reporter assay reagent (Promega) according to the manufacturer's instructions. The results are given as the mean fold change in ARE promoter activation ± standard deviation (SD) from at least three independent experiments. Where indicated, recombinant protein expression was verified by immunoblotting on 50 μg of total cell lysates by using anti-HA and anti-FLAG-M2 mouse monoclonal antibodies (Merck Life Sciences) and anti-actin as a loading control (Merck Life Sciences).

### Subcellular fractionation

HEK-293T cells expressing wt-ORF6 or the corresponding variants are collected at 24 h post-transfection. Pure nuclear and cytoplasmatic fractions are obtained by using NE-PER Nuclear and Cytoplasmic Extraction Reagents (Pierce, Milan, Italy) following the manufacturer’s instructions. Cytoplasmic and nuclear fraction protein content was determined by BCA protein assay kit (Pierce, Milan, Italy) and stored at − 80 °C for further use.

### Immunoblotting

A total of 50 µg of total proteins was boiled in Laemmlie sample buffer for 5 min, separated by SDS‒PAGE and transferred to a NitroBind nitrocellulose membrane (Santa Cruz Biotechnology, Heidelberg, Germany). After blocking with Intercept Blocking Buffer (LI-COR Biosciences, Germany), antioxidant proteins are detected following membrane incubation with anti-G6PD, anti-NRF2 (Cell Signaling Technologies, Milan, Italy), anti-SOD1, anti-HO-1 (Santa Cruz Biotechnologies, Milan, Italy), anti-p38 and phosphorylated p38 (Santa Cruz Biotechnologies, Milan, Italy), anti-phosphorylated NRF2 (Ser40) (Thermo Fisher Scientific, Milan, Italy), anti-PKC (Santa Cruz Biotechnologies, Milan, Italy) or anti GSK-3β (Santa Cruz Biotechnologies, Milan, Italy). Actin was used as a loading control. After being washed three times with PBS containing 0.05% Tween-20 (PBS-T), the membranes are incubated with IRDye800/680-labelled goat anti-mouse/rabbit secondary antibodies (LI-COR Biosciences, Germany). Membranes are analysed by Odyssey Infrared Imager, and integrated intensities of fluorescence are used for densitometry analysis by using ImageJ software.

### Quantitative assessment (RT‒qPCR) of oxidative stress markers expression

A549 and HEK-293T cells are transfected with either empty vector or plasmids expressing wild-type or mutant ORF6 variants as previously described. Cells are collected 24 h later, and total cellular RNA was isolated using the RNAeasy PLUS mini kit (Qiagen, Milan, Italy) following the manufacturer’s recommendations. Afterwards, the quality and quantity of the extracted RNA was verified by spectrophotometric reads, and equal amounts of RNA are subjected to a Reverse-Transcription quantitative PCR (RT‒qPCR) with SensiFAST reversion transcription kit and SensiFAST SYBR No-ROX PCR kit (Bioline) for cDNA synthesis and Nuclear factor erythroid 2-related factor 2 (NRF2), Glucose-6-phosphate dehydrogenase (G6PD) and Heme Oxygenase 1 (HO-1) gene expression analysis. Specific primers are available upon request. 18S rRNA was used as an internal control for amplification and normalization. The fold change in specific mRNA content was calculated relative to the mock, empty plasmid, transfected cells by the 2^−∆∆Ct^ algorithm. The results are provided as the mean fold change from at least four independent experiments ± standard deviation.

### Glutathione assay

GSH levels are quantified in transfected A549 cells collected at 24 h and 48 h post-transfection by using the Glutathione Assay Kit (Enzo Life Sciences, Milan, Italy) following the manufacturer’s instructions. Total GSH was quantified in cell lysates after deproteinization with metaphosphoric acid. Briefly, an aliquot of deproteinized samples was first incubated with 2-vinylpyridine to derivatize GSSG. Reduced GSH levels are calculated by differences between total GSH and GSSG and normalized to the protein content of each sample determined by the Bradford method (Bio-Rad, Milan, Italy). The results are provided as the mean fold change in GSH content ± standard deviation from at least three independent experiments.

### Measurement of the intracellular ROS level

2′,7′-Dichlorofluorescin diacetate (H2DCFDA)-based (Merck Life Sciences) assays are used to detect the accumulation of intracellular reactive oxygen species (ROS) in A549 transfected cells. Briefly, A549 cells mock-transfected or expressing the wild-type or mutated ORF6 proteins are treated at 48 h post-transfection with 50 μM DCFDA or vehicle diluted in complete phenol red-free culture medium for 30 min at 37 °C. After extensive washes with phosphate-buffered saline (PBS), the generation of highly fluorescent 2',7'-dichlorofluorescein (DCF) was evaluated by microscopy (Eclipse Ts2, Nikon, Florence, Italy). For quantitative detection of DCF, fluorescence intensities are measured at 485/535 nm (excitation/emission) in a VICTOR™3 microplate reader (Perkin Elmer).

### Statistics

The mean differences are statistically analysed by means of Mann Whitney U test among different study groups. Statistical significance was set at *p* < 0.05, two-tailed.

## Results

### SARS-COV-2 ORF6 modulates intracellular ROS production

**Fig. 1:**
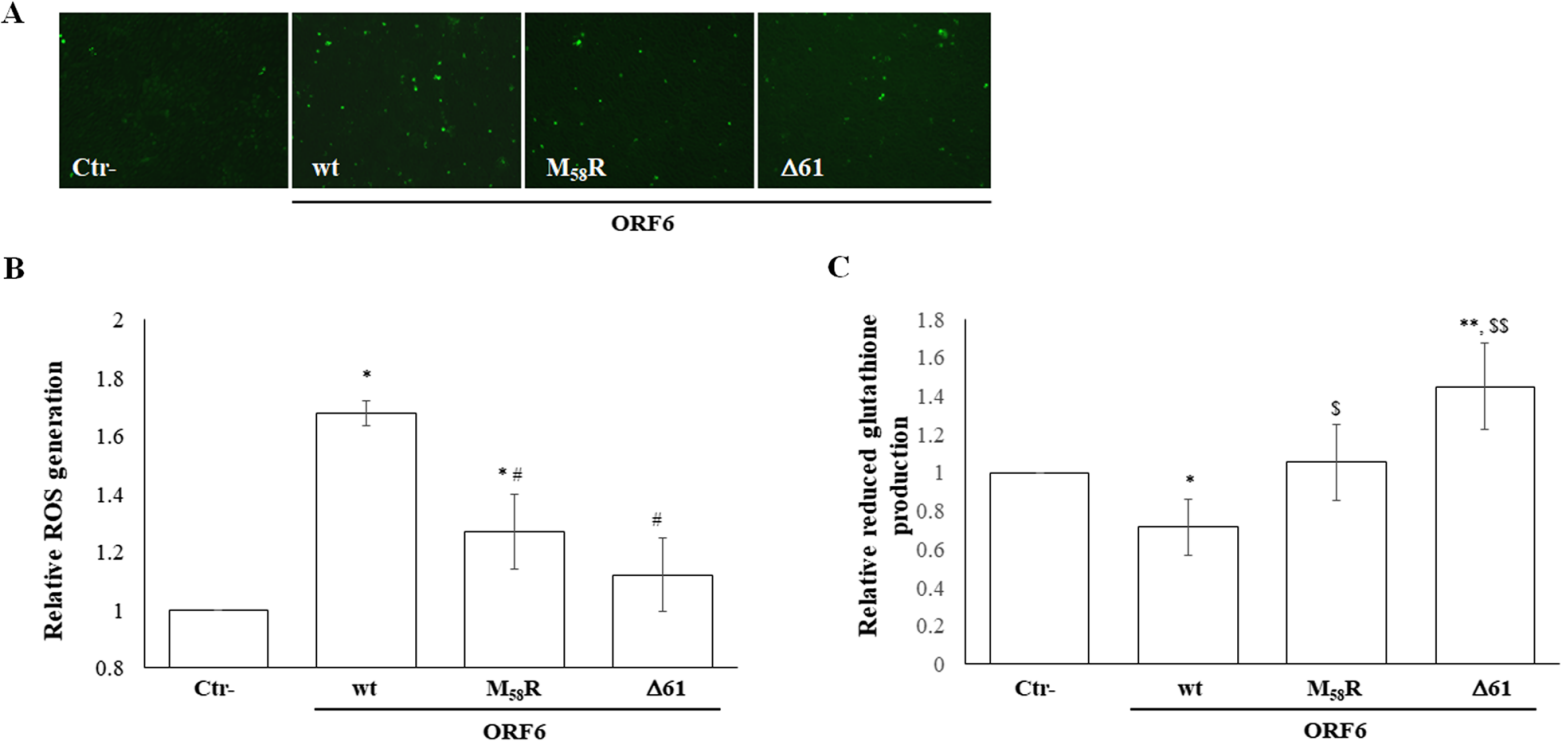
SARS-CoV-2 ORF6 protein induces cellular stress. A549 cells ectopically expressing wild-type (wt)-ORF6 or the M_58_R and D61 mutants are stained with the reduced, nonfluorescent form of fluorescein (H_2_DCFDA) as an indicator for Reactive Oxygen Species (ROS). Upon cleavage of the acetate groups by intracellular esterases and oxidation, the highly fluorescent 2',7'-dichlorofluorescein (DCF) is determined by (**A**) fluorescence microscopy and (**B**) by microplate reding (Ex:485/Em:535) on cell lysates. The results are presented as the mean fold change in fluorescence intensities ± standard deviations (SD) from at least three (*n* > 3) independent experiments, each condition tested in duplicate. The same samples are analysed for (**C**) reduced glutathione (GSH) levels. The differences between total GSH and its oxidized form (GSSG), normalized to the protein content of each sample, are calculated. The results are provided as the mean fold change in GSH content ± standard deviation (SD) from three (*n* = 3) independent experiments. Significance is determined with respect to the negative control (Ctr-), transfected with empty plasmid, sample as *p* < 0.005, ^**^; *p* < 0.05, ^*^; or to the wt-ORF6 variant as *p* < 0.05, ^#;$^, *p* < 0.005, ^$$^

SARS-CoV-2 has been reported to trigger Reactive Oxygen Species (ROS) production during viral infection [44]. However, most research has focused on humans and animal in vivo models to evaluate altered cellular redox state during acute infection, frequently associated with severe clinical profiles. Conversely, little effort has been made to identify viral protein(s) involved in controlling these pathways. Therefore, we investigated whether the SARS-CoV-2 ORF6 protein, known for its antagonistic effect on several cellular processes, has the ability to modulate ROS production. In parallel, we also investigated the impact of ORF6 mutants to identify functional domains within the protein. Indeed, as previously demonstrated, the ORF6 C-terminus has a critical role in protein function. Therefore, the naturally occurring ORF6 mutant carrying the deletion of the last amino acid (ORF6-Δ61) and the methionine 58 mutant (M_58_R) ORF6 variants were included in the study [51, 52]. The impact of the ORF6 viral protein on intracellular ROS production was determined by utilizing the fluorescent dye DCF. A549 cells ectopically expressing wt-ORF6 or its mutant variants were collected 24 h post-transfection and then stained with H_2_DCFDA, a nonfluorescent molecule that becomes fluorescent (DCF) in the presence of ROS due to their reducing activity. Fluorescence microscopy examination of transfected cells shows that, compared to the empty plasmid transfected control, the DCF fluorescence is increased in cells expressing wt-ORF6. In contrast, cells expressing the M_58_R and the Δ61 mutants show minor, non-significant, variations in DCF production (Fig. 1A). These findings suggest that the ORF6 protein is associated with intense ROS generation, achieved by suppressing the cellular scavenger system. To better quantify the differences in ROS generation caused by the presence of ORF6 variants, the release of DCF was quantified by fluorescence measurements (Ex/Em: ∼492–495/517–527 nm) of the cell suspension. As reported in Fig. 1B, a mean fold increase of 1.68 ± 0.04 (*p* = 0.046) and 1.27 ± 0.13 (*p* = 0.021) is observed in cells ectopically expressing wt and M_58_R ORF6, respectively, whereas the presence of Δ61 protein variant does not affect cellular ROS content (fold change; 1.12 ± 0.12, *p* = 0.642). To further support the evidence that ORF6 is associated with the induction of oxidative stress, intracellular reduced glutathione (GSH) was quantified in transfected A549 cells. As reported in Fig. 1C, the presence of wt-ORF6 protein counteracts the physiological cellular response to the increased ROS at 24 h post-transfection, leading to a significant reduction in GSH of approximately 30% (mean fold change 0.71 ± 0.15; *p* = 0.02). In contrast, the expression of the M_58_R ORF6 mutant is not associated with altered intracellular GSH content (1 ± 0.19; *p* = 0.063), while Δ61 mutant lead to the activation of the GSH-based detoxifying cellular system (mean fold change 1.45 ± 0.22; *p* = 0.002) (Fig. 1C).

### SARS-CoV-2 ORF6 hinders NRF2 activity to suppress the antioxidant response

**Fig. 2:**
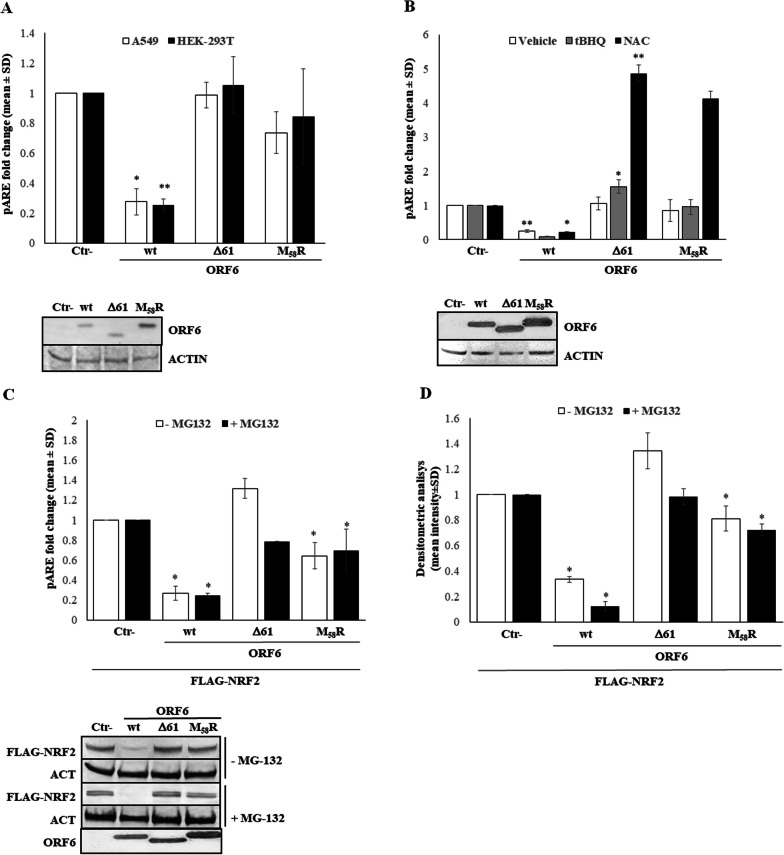
The activation of Antioxidant-Responsive Element (ARE)-containing promoter is differentially modulated by ORF6 protein variants*.* The activity of different SARS-COV-2 ORF6 protein mutants towards the ARE promoter is investigated in (**A**) A549 and HEK-293T cells transfected with ARE-mediated firefly luciferase (pARE) and SV40 promoter-mediated *Renilla* luciferase reporter plasmids along with ORF6-expressing plasmids or empty vector (Ctr-). In parallel, (**B**) transfected HEK-293T cells are vehicle-treated and treated with tert-Butylhydroquinone (tBHQ) or N-acetyl cysteine (NAC) at 36 h post-transfection. A pARE activation reporter assay (**C**) is conducted in HEK-293T cells over-expressing ectopic NRF2 (FLAG-NRF2) along with ORF6-expressing plasmids or empty vector (Ctr-) and exposed to the proteasome inhibitor MG-132 or to vehicle. At 48 h post-transfection, cells are collected and luciferase activities are measured. Meanwhile, variations in FLAG-NRF2 protein content are assessed by immunoblotting (**D**) on lysates of vehicle- or MG-132-treated transfected samples. Densitometric analysis is performed and the results are plotted as the mean fold change in target proteins with respect to the empty plasmid-transfected control from three (*n* = 3) independent experiments ± standard deviations (SD). Significance is determined with respect to the negative control (Ctr-), transfected with empty plasmid, sample as **p < 0.005; *p < 0.05

The transcription factor Nuclear factor erythroid 2-related factor 2 (NRF2) plays a key role in activating the antioxidant response to counteract oxidative stress stimuli and ROS production occurring during viral infections. The recruitment of NRF2 at the Antioxidant Response Element (ARE) located in the promoter of many human genes, particularly those responsible for scavenging ROS, is essential for the induction of antioxidant enzymes and the establishment of an efficient detoxifying response. Studies conducted on Influenza A virus have shown the downregulation of NRF2 protein levels or its activation and the overproduction of ROS during infections [30]. Similarly, SARS-CoV-2 infection has been associated with a down-regulation of NRF2, although this mechanism has not been deeply investigated [34–36, 47, 48]. Using a reporter assay in which the expression of the luciferase reporter gene is a consequence of ARE *cis*-element activation, we tested the antagonistic activity of the SARS-CoV-2 ORF6 protein on the NRF2 signaling pathway. In A549 epithelial lung cells, the ectopic overexpression of the wt-ORF6 protein concurs with the dramatic reduction in ARE-mediated luciferase expression, leading to a 3.6-fold reduction (fold change 0.27 ± 0.088; *p* = 0.019) in specific promoter activation (Fig. 2A). In contrast, the M_58_R ORF6 mutant, previously described as inactive towards nucleopore proteins hindrance, lose its antagonistic function on NRF2/ARE axis activation, with markedly reduced antagonistic activity (0.73 ± 0.13-fold promoter activation, *p* = 0.059) compared to the negative control (Fig. 2A). On the other hand, ORF6-Δ61 is completely incapable of hindering ARE promoter activation (fold change 0.98 ± 0.08; *p* = 0.642) (Fig. 2A). Similar results are obtained when we used the HEK-293T cell line to assess pARE activation. In this cell system, only wt-ORF6 demonstrates the ability to mediate repression of the indicated promoter, resulting in approximately a four-fold reduction in pARE activation (fold change 0.25 ± 0.04; *p* = 0.002), as depicted in Fig. 2A. To further support the antagonistic effects of the ORF6 protein on NRF2 signaling, its inhibitory activity towards ARE-containing promoter activation was investigated in a NRF2-stimulated system in which the antioxidant tert-Butylhydroquinone (tBHQ), which targets Keap1, and N-acetyl-cysteine (NAC), a well-known antioxidant compound, resulted in NRF2 hyper-activation. Since A549 alveolar epithelial cells present a mutation in the Keap1 gene, this cell system is insensitive to tBHQ and NAC [84]; thus, HEK-293T cells were used. The wt-ORF6 strongly counteracts both the tBHQ and NAC-inducible functions, reducing pARE activity by 12- and 4.8-fold, respectively (*p* = 0.006 and *p* = 0.02, respectively) (Fig. 2B). Conversely, the presence of the M_58_R mutant does not alter the tBHQ activity (fold change 0.95 ± 0.21; *p* = 0.877), while the Δ61-ORF6 variant exerts a costimulatory function on NRF2, leading to an up-regulation of pARE activity of 1.5-fold (*p* = 0.007) (Fig. 2B). Surprisingly, both ORF6 mutants have synergistic effects with NAC on the NRF2-ARE axis, since the antioxidant properties of the drug are augmented by more than four fold (*p* < 0.005) by viral protein expression (Fig. 2B). To address whether this antagonistic function is a consequence of ORF6 activity on newly synthesized mRNA movements to the cytoplasm, thus limiting cellular protein translation, or to a target-specific activity of the transcription factor, the pARE reporter assay was then assessed in HEK-293T cells over-expressing ectopic FLAG-tagged NRF2. Despite the small amount of transfected FLAG-NRF2 plasmid, the resulted protein sufficiently accumulates into the cells, as demonstrated by immunoblotting by using anti-FLAG epitope antibody (Fig. 2C, lower panel), and strongly stimulates ARE promoter activation, leading to a 16.4 ± 4.1 (p < 0.005) folds increase in pARE-mediated firefly luciferase expression (data not shown). The antagonistic property of wt- and M_58_R ORF6 on NRF2 is evidenced by a drastic decrease in the ectopic transcription factor content (Fig. 2C, lower panel) and, consequently, in pARE activation (fold change 0.26 ± 0.07; *p* = 0.021 and 0.64 ± 0.13; *p* = 0.02, respectively) (Fig. 2C). In contrast, and in agreement with previously results, the Δ61-ORF6 mutants is unable to counteract NRF2-mediated specific promoter activation even when the transcription factor is over-expressed (fold change 1.3 ± 0.47, *p* = 0.278) and its cellular accumulation is not perturbed (Fig. 2C). To examine the effects of ORF6 on NRF2 viability, we studied transcription factor activity in MG132-treated cells. Indeed, since NRF2 function is limited by Keap1 interaction and its subsequent ubiquitination and degradation, the MG-132 proteasome inhibitor behaves as an antioxidant increasing NRF2 cytoplasmic accumulation. Although proteasome inhibition by MG-132 is associated with an increase in NRF2 accumulation, perturbation of cellular homeostasis is also observed. However, in HEK-293T cells expressing the wt- or the M_58_R mutant ORF6 protein, the antagonistic behaviour of the viral protein towards pARE activation is not abolished by MG-132 (fold change 0.24 ± 0.018; *p* = 0.019, 0.69 ± 0.021; *p* = 0.039 respectively) (Fig. 2C). Instead, the activity of the Δ61 mutated ORF6 variant is not altered (*p* = 0.059) by the presence of the inhibitor. Furthermore, immunoblotting detection of ectopic FLAG-tagged NRF2 shows that its expression is significantly decreased in wt and, to a lesser extent, in M_58_R ORF6-expressing HEK-293T cells despite MG-132 treatment (fold change 0.12 ± 0.04; *p* = 0.019, 0.72 ± 0.04; *p* = 0.039) (Fig. 2D). In contrast, NRF2 protein is equally accumulated in the cytoplasm of control cells and ∆61-ORF6 variant-containing cells (Fig. 2D). It's important to note that, while MG-132 treatment is expected to increase protein accumulation by reducing their turnover through the ubiquitin–proteasome system, the drug may also have broader effects on general protein translation and cell proliferation. This is evident from the observed decrease in NRF2 content in all samples exposed to MG-132. However, these results further underline the activity of the viral protein on the NRF2 translational process rather than on mature NRF2 protein turnover.

### Transcriptional control of the NRF2 pathway is not impaired by ORF6

**Fig. 3:**
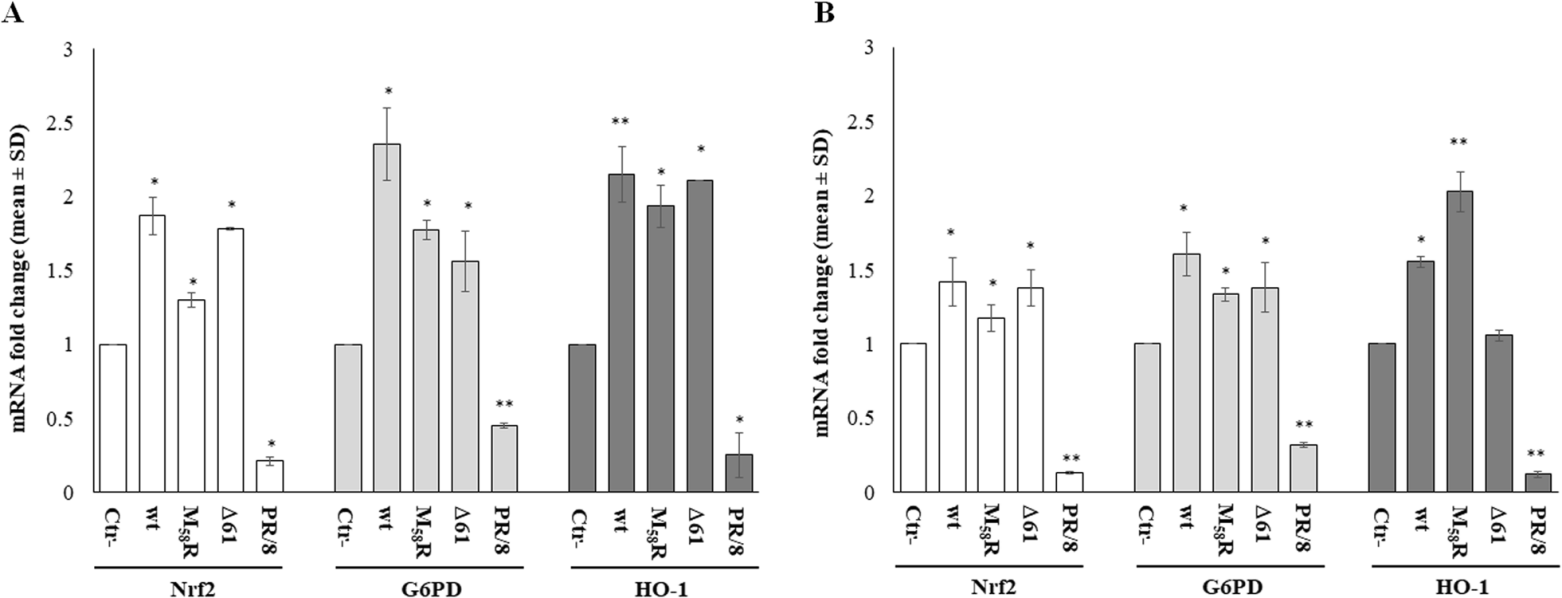
Cellular antioxidant response is induced by ORF6 at the transcriptional level. The effects of SARS-CoV-2 ORF6 protein, wt or mutated forms, on cellular antioxidant modulation are evaluated at the transcriptional level in (**A**) HEK-293T and (**B**) A549 cells. Total RNA is purified from empty plasmid (Ctr-) or ORF6-transfected cells collected at 24 h post-transfection. Specific Nuclear factor erythroid 2-related factor 2 (NRF2), Glucose-6-Phosphate Dehydrogenase (G6PD) and Heme Oxygenase-1 (HO-1) mRNA levels are detected by quantitative reverse-transcription polymerase chain reaction (RT‒qPCR). 18S gene expression is used for relative quantification based on the 2^−ΔΔCt^ method. Data are expressed as mean values ± standard deviations (SD) of at least three (*n* ≥ 3) independent experiments, each performed in duplicate. Significance is determined with respect to the negative control (Ctr-), transfected with empty plasmid, sample as **p < 0.005; *p < 0.05

The transactivation of promoters containing the ARE consensus sequence and antioxidant gene expression is regulated by binding of the transcription factor NRF2 to the mentioned promoter regions [37]. Based on our preliminary results, to further investigate whether ORF6 impacted the antioxidant cellular response mediated by NRF2, we quantified NRF2 mRNA in HEK-293T cells, harbouring an intact antioxidant system, after ectopic expression of the viral protein. RT‒qPCR analysis demonstrates that NRF2 mRNA expression is induced by wt or mutant ORF6 at 24 h post-transfection. The ectopic expression of wt-ORF6 induces a significant increase (*p* = 0.008) of almost twofold (1.9 ± 0.13) in NRF2 mRNA accumulation, whereas the M_58_R mutated protein leads to a modest (1.3 ± 0.05-fold induction) (*p* = 0.01) increase in NRF2-specific transcripts. Similarly, Δ61-ORF6 upregulates NRF2 expression at the transcriptional level (fold stimulation 1.8 ± 0.007; *p* = 0.019) (Fig. 3A). These data are further validated by evidence that Influenza virus PR/8 infection of cultured HEK-293T cells results in a transcriptional downregulation of NRF2 at early times of infection (24 h), leading to a decrease (fold expression 0.21 ± 0.03; *p* = 0.006) in specific mRNA content, as previously demonstrated in literature [30] (Fig. 3A). Accordingly, NRF2 modulation by ORF6 variants influences the expression of scavenger proteins, such as Glucose-6-Phosphate Dehydrogenase (G6PD) and Heme Oxygenase-1 (HO-1). Indeed, both detoxifying genes are significantly upregulated at the transcriptional level (*p* < 0.05) (fold change ~ 2 ± 0.22) by wt-ORF6 and both protein mutants (Fig. 3A). Moreover, Influenza virus PR/8 infection mediates 0.45 ± 0.01 (*p* = 0.005) and 0.25 ± 0.15 (*p* = 0.029) fold reduction in G6PD and HO-1 scavenger’s gene expression, respectively (Fig. 3A). The described ORF6 activity is further reported when the ectopic expression of the viral proteins is accomplished in A549 cells, although lower expression of the foreign proteins is observed in this cell line. Indeed, despite A549 transfected cells are widely used in in vitro experimental models of SARS-CoV-2 infection [53], the expression of NRF2-controlled genes might not reflect the physiological condition because of their Keap1 mutation [54]. Notwithstanding, this cell line has been used in several studies concerning redox state changes during viral infections, including ours [30]. In this study, we observe a significant induction of the NRF2 axis due to the presence of ORF6, wt- or mutated versions (Fig. 3B).

### NRF2 is downregulated by ORF6 to maintain oxidative stress conditions

**Fig. 4:**
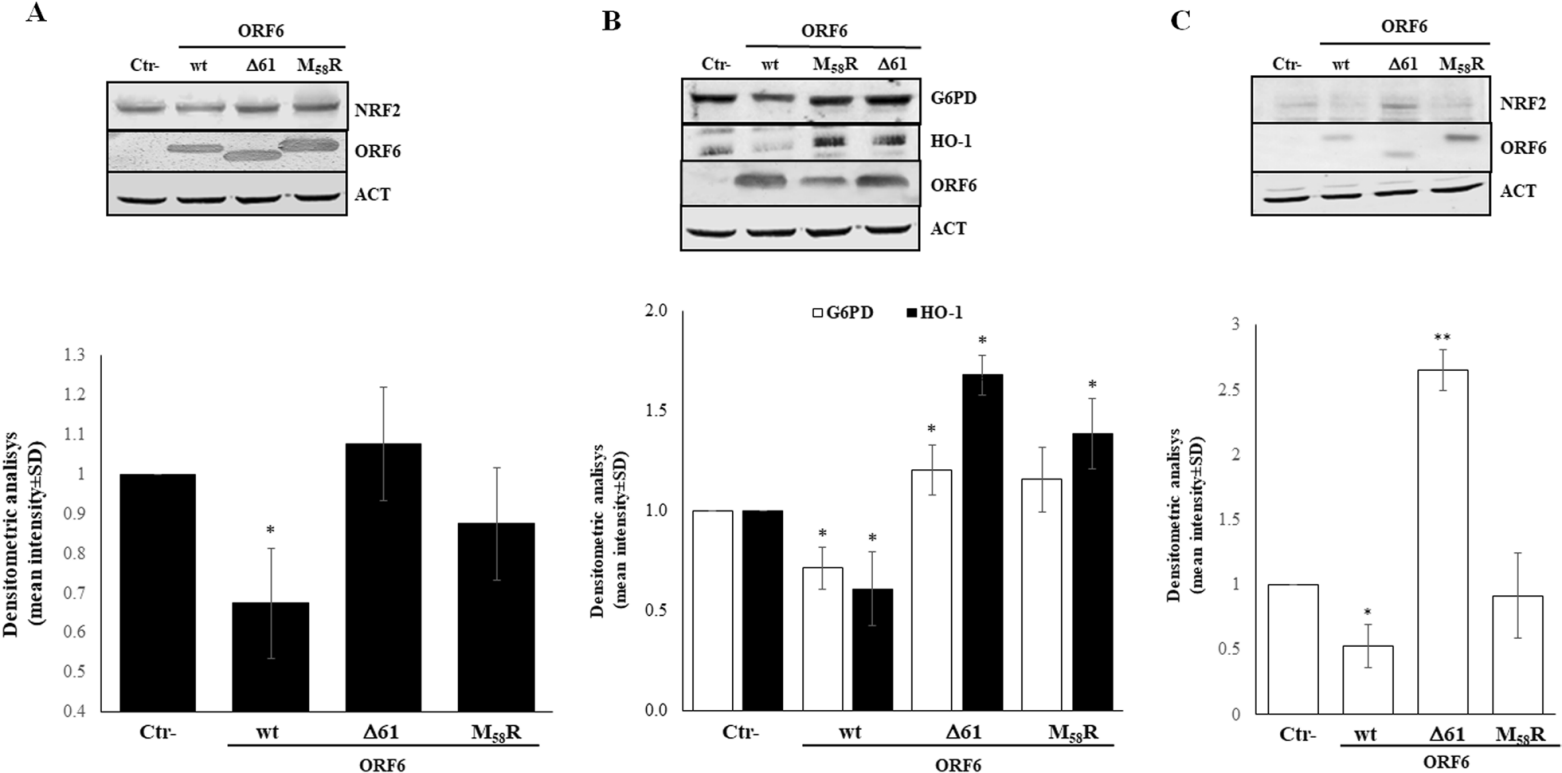
ORF6 protein hinders scavenger protein content. The effect of SARS-CoV-2 ORF6 protein, wt- or mutated variants, on the cellular antioxidant response is further analysed by immunoblotting for (**A**) NRF2 and (**B**) G6PD and HO-1 on 50 μg of whole cell lysates (WCL) of transfected HEK-293T cells. (**C**) The effects of ORF6 protein mutants on endogenous NRF2 expression is further investigated on WCL of A549 cells expressing ORF6 protein variants. The loading control is represented by immuno-detection of actin protein. Densitometric analysis of reactive bands is performed by ImageJ software and the results are plotted as the mean fold change in target proteins, normalized with respect to relative actin levels, from (*n* = 3) independent experiments, performed in duplicate, ± SD. Significance is determined with respect to the negative control (Ctr-), transfected with empty plasmid, sample as **p < 0.005; *p < 0.05

Previous results showed that the infection of both Calu-3 and Vero E6 cells by SARS-CoV-2 determines a downregulation of signaling proteins involved in cellular homeostasis and the antioxidant scavenger system [33, 35]. Olagnier and colleagues identified NRF2 as the major factor affected by virus infection and replication, leading to the suppression of NRF2-inducible proteins HO-1 and NAD(P)H quinone oxidoreductase-1 (NqO-1) [34]. Based on these evidences, we further analysed the expression pattern of antioxidant-related genes upon SARS-CoV-2 ORF6 ectopic expression in HEK-293T and A549 cells. In contrast to the mRNA expression profile reported above, the antioxidative response is significantly suppressed by the presence of wt-ORF6 at the protein level. We performed an immunoblot assay on HEK-293T whole-cell lysates (WCLs) to assess NRF2, G6PD, and HO-1 protein levels after SARS-CoV-2 ORF6 expression. We find that overexpression of viral wt-ORF6 significantly reduces endogenous NRF2 protein content by approximately 40% (*p* = 0.007), whereas neither the M_58_R (mean fold 1.16 ± 0.88, *p* = 0.11) nor the Δ61-ORF6 (mean fold 1.09 ± 0.16, *p* = 0.64) presence perturbs the physiological levels of NRF2 protein within the cell (Fig. 4A). In addition, the antioxidant effectors G6PD and HO-1, whose expression is regulated by NRF2, are markedly reduced (fold change 0.71 ± 0.10, *p* = 0.03; 0.61 ± 0.18, *p* = 0.012, respectively) when wt-ORF6 is expressed in HEK-293T cells (Fig. 4B). Instead, both M_58_R and ORF6-Δ61 have no significant impact on G6PD and HO-1 cytoplasmic accumulation (Fig. 4B), suggesting that the amino acid at the ORF6 C-terminus (D61) is involved in the observed NRF2 protein downregulation. To further confirm our findings, NRF2 protein expression was also assessed in A549 cells. In the new cellular system, a pronounced decrease in the transcription factor is observed when wt-ORF6 (fold change 0.52 ± 0.16; *p* = 0.012) is expressed. However, M_58_R does not display detectable activity toward NRF2 (fold change 0.95 ± 0.32; *p* = 0.08) (Fig. 4C). Conversely, the Δ61-ORF6 variant is completely inactive in down-regulating NRF2 protein content in A549 cells (fold change 2.6 ± 0.15; *p* = 0.005) (Fig. 4C).

### NRF2 nuclear movement is hindered by ORF6

**Fig. 5:**
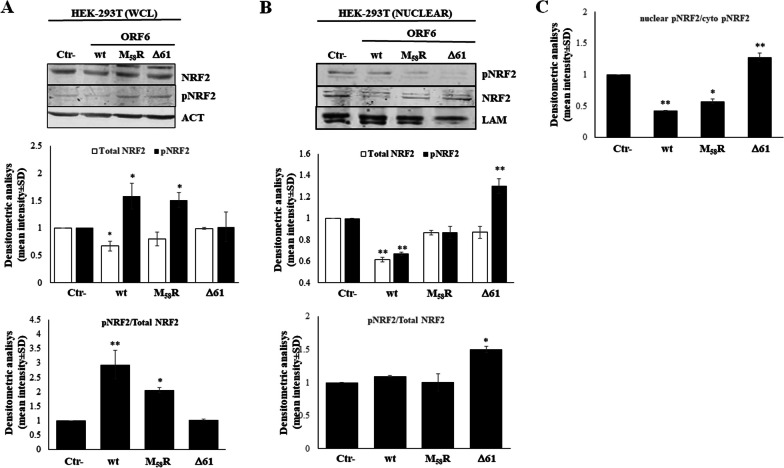
ORF6 hinders NRF2 nuclear translocation but not its activation by phosphorylation. NRF2 activation is assessed by immunoblotting analysis on whole cell lysate of (**A**) HEK-293T cells expressing the wt-ORF6, M_58_R or ∆61 protein mutants for total NRF2 or serine 40 phosphorylated NRF2 (pNRF2) expression (mid panel). Densitometric analysis of reactive bands is performed by ImageJ software and, after actin normalization, the fold change in protein accumulation is calculated. The activation rate of NRF2 is inferred by the pNRF2/total NRF2 ratio (lower panel). Nuclear translocation of NRF2 (**B**), either total or pNRF2 (mid panel), is investigated in HEK-293T cells expressing the wt-ORF6, M_58_R or ∆61 proteins by immunoblotting. Lamin A/C is used as a loading control. Densitometric analysis is performed on data obtained from three independent experiments, and each condition is run in duplicate (*n* = 6). Graph values are expressed as the mean fold change in band intensities ± standard deviations (SD). The ratio of nuclear to cytoplasmic pNRF2 is shown in (**C**), evidencing the reduced nuclear translocation of the active transcription factor. Significance is determined with respect to the negative control (Ctr-), transfected with empty plasmid, sample as **p < 0.005; *p < 0.05

**Fig. 6:**
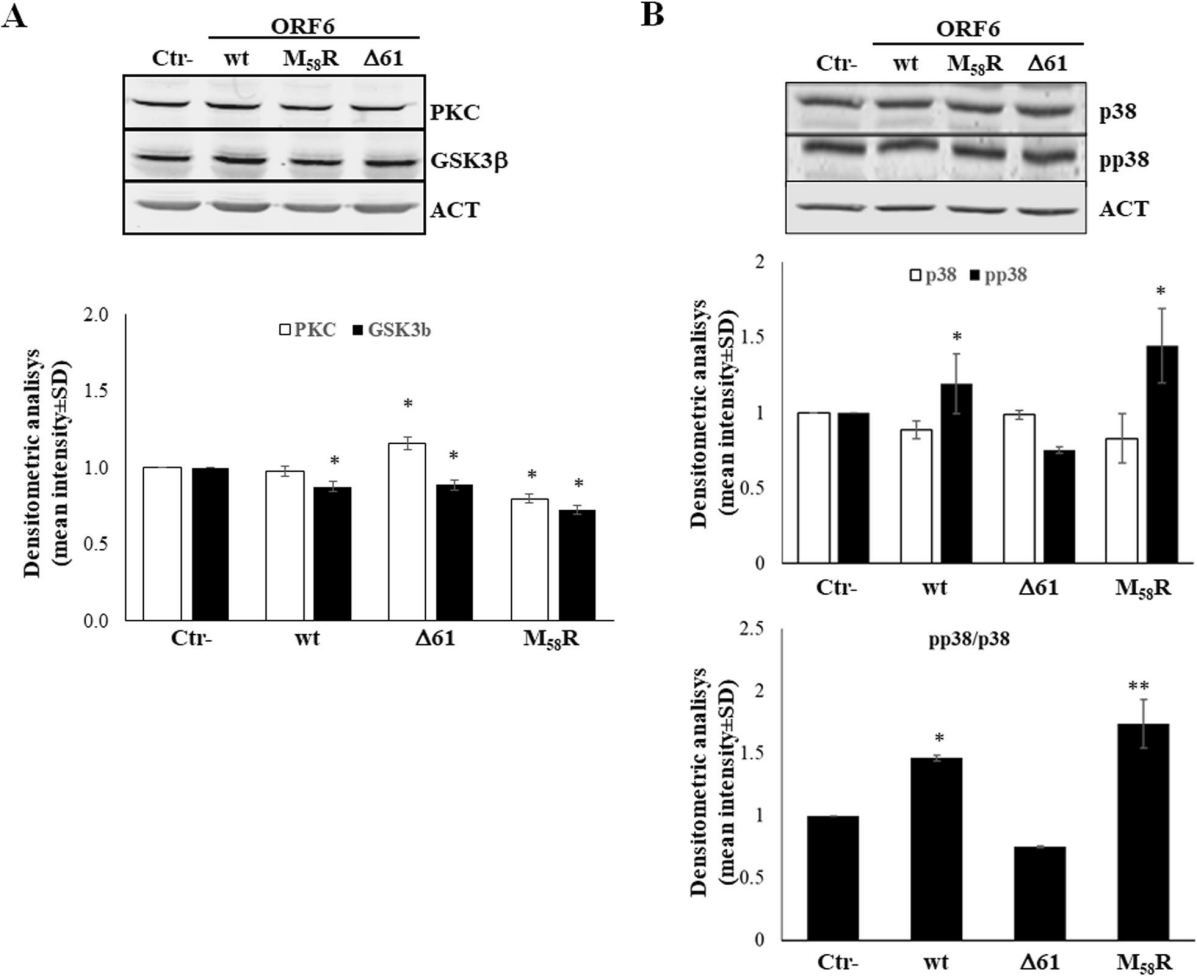
ORF6 controls p38 MAPK activity on NRF2 phosphorylation. The cytoplasmic content of (**A**) Protein Kinase C (PKC) and Glycogen Synthase Kinase 3-beta (GSK-3b) or (**B**) p38 Mitogen-Activated Protein Kinase (MAPK), total or phosphorylated (pp38), is investigated in HEK-293T cells expressing either wt-ORF6 or the previously described mutant versions. Densitometric analysis of immunoreactive protein bands is performed by ImageJ software by normalization with relative actin loading controls. The results are plotted as the mean fold change in target proteins with respect to the empty plasmid-transfected control from three (*n* = 3) independent experiments ± standard deviations (SD). Significance is determined with respect to the negative control (Ctr-), transfected with empty plasmid, sample as ***p < 0.0005; **p < 0.005; *p < 0.05

Based on our evidence demonstrating the downregulation of NRF2 protein mediated by wt-ORF6 and the M_58_R mutant, we further investigated NRF2 activation in the HEK-293T cell system. While NRF2 activity is mainly regulated by its turnover through Keap1-mediated ubiquitination and proteasomal degradation, it can also be regulated at the post-translational level. Phosphorylation at the serine, threonine or tyrosine residues in NRF2 may affect, both positively or negatively, the process of NRF2 proteasomal degradation, nuclear movement and binding to the ARE-containing promoter sequence [54]. In the present study, we mainly focused on NRF2 serine 40 (S_40_) phosphorylation since it has been reported to promote NRF2 dissociation from Keap1, thereby leading to increased nuclear translocation and transcription of ARE-driven antioxidant genes [55]. The immunoblotting performed on whole cell lysates of transfected HEK-293T cells shows a significant increase in S_40_ phosphorylated NRF2 (pNRF2) when both wt and M_58_R ORF6 are expressed (1.58 ± 0.23; *p* = 0.012, 1.51 ± 0.22; *p* = 0.012), in line with the previously described increase in ROS cytoplasmic accumulation, whereas the ∆61 mutant lead to a marginal, non-significant (1.02 ± 0.15, *p* = 0.083), activation of the transcription factor (Fig. 5A, upper panel). Data analysis based on the ratio between phosphorylated and total NRF2 content clearly shows a strong increase in pNRF2 when the wt and M_58_R ORF6 variants are present (2.9 ± 0.49; *p* = 0.002, 1.9 ± 0.29; *p* = 0.012), while the ∆61 protein fails to induce NRF2 activation (1.02 ± 0.2; *p* = 0.22) by phosphorylation (Fig. 5A, lower panel). Afterwards, the possible ORF6 hindrance of NRF2 nuclear translocation was assessed in nuclear fractions of HEK-293T cells expressing the wt-ORF6, M_58_R or ∆61 protein. As reported in Fig. 5B, wt-ORF6 only determines a decrease in nuclear NRF2 levels (fold change 0.61 ± 0.02; *p* = 0.005), which, consequently, is associated with a decrease in its phosphorylated form in the same cellular compartment (fold change 0.67 ± 0.01; *p* = 0.002) (Fig. 5B). In contrast, both the M_58_R and ∆61 ORF6 mutant proteins lead to marginal (fold change 0.86 ± 0.02; *p* = 0.22 and 0.87 ± 0.05; *p* = 0.22) variation in nuclear NRF2 content. Among them, only the ORF6-∆61 mutant is able to induce an increase in pNRF2 nuclear accumulation (1.3 ± 0.07; *p* = 0.002), which is better evidenced by the ratio between nuclear pNRF2/nuclear NRF2 (1.5 ± 0.04; *p* = 0.019). These results suggest that wt-ORF6 do not impair NRF2 activation by phosphorylation but rather down-regulates NRF2 accumulation in both nuclear and cytoplasmic compartments. Moreover, the reduced nuclear localization of pNRF2 compared to the cytoplasmic counterpart detected in both wt and M_58_R ORF6-expressing cells (0.42 ± 0.08; *p* = 0.005, 0.57 ± 0.03; *p* = 0.012) also confirms the hindrance by the viral protein of nuclear movement of the indicated transcription factor upon its cytoplasmic activation (Fig. 5C). As supporting evidence, the deleted ORF6 does not hinder pNRF2 translocation, since a 1.27 ± 0.07-fold increase (*p* = 0.005) in nuclear, active, NRF2 is noticed (Fig. 5C). HEK-293T transfected samples were further analysed to monitor the cellular levels of kinases that are known to be activated by oxidative stress stimuli and are involved in NRF2 modulation: Protein Kinase C (PKC), Glycogen Synthase Kinase 3-beta (GSK-3β), and p38 mitogen-activated protein kinases (MAPKs). Contrary to PKC activity, GSK-3β is observed to phosphorylate NRF2, inducing Cullin-1/Rbx1-mediated NRF2 ubiquitination and subsequent degradation [56], while the p38 MAPK has been reported to both positively and negatively regulate the NRF2-mediated antioxidant response [57–61]. In our hands, the ORF6 reveals marginal control, although statistically significant, on PKC and GSK-3β cellular content (Fig. 6A). On the contrary, we observe that activated phosphorylated p38 (pp38), relative to total p38 protein level, is markedly increased in samples where wt-ORF6 (fold change 1.43 ± 0.02, *p* = 0.031) or M_58_R (fold change 1.73 ± 0.19, *p* = 0.005) is present, whereas the ∆61 protein variant is unable to upregulate kinase activity (fold change 0.75 ± 0.005, *p* = 0.063), suggesting a role for MAPKs in ORF6 activity toward NRF2 (Fig. 6B). These data agree with previous data showing NRF2 hyper-phosphorylation in both M_58_R- and wt-ORF6-expressing cells, while unperturbed pNRF2 levels are described for ∆61-ORF6 (Fig. 5A).

## Discussion

There are many evidences that SARS-CoV-2, similar to its ancestors SARS-CoV and MERS-CoV, has evolved multiple mechanisms to overcome cellular pathways, including innate immunity and cellular oxidative stress, to elicit virus replication [16, 17, 44, 62, 63]. Some authors have depicted the importance of the antioxidant response during SARS-CoV-2 infection, both in vitro and in vivo, demonstrating the Nuclear factor erythroid 2-related factor 2 (NRF2) downregulation during viral infection [34]. Furthermore, Zhang et al*.* proposed the involvement of the NSP14 viral protein in the NRF2 pathway to control the cell response to infection [48]. Within virus accessory proteins, ORF6 has an important role in host immunity evasion by inhibiting the nuclear translocation of STAT1 and hindering nucleopore traffic of newly synthetized mRNAs to block IFN-β and other cytokines (*e.g.,* IL-6) expression [51, 52, 64]. SARS-CoV-2 variants differ from the Wuhan-1 strain by genetic mutations, mostly located in the Spike protein, but also other viral proteins are affected by virus genetic evolution, determining mutations or deletions in both structural and non-structural proteins [65–68]. Mutations outside the Spike protein are likely to contribute to virus pathogenicity, such those targeting the ORF6 which, by enhancing nuclear retention of newly synthetized mRNAs, allow the virus to escape from the immune system [51, 52, 64, 66–68]. Among them, the ORF6 D_61_L substitution described for BA.2 and BA.4 Omicron sub-lineages influences viral protein function. Indeed, while the methionine 58 mutation (M_58_R), described as inactive with respect to the nucleopore complex hindrance, partially reverts the antagonistic properties of ORF6, the D_61_ deletion (ORF6-∆61) completely abrogated protein function. Therefore, the ORF6 impact on NRF2, which represents the master transcription factor involved in the cell antioxidant response and inflammation, deserves to be evaluated. In the present study, we demonstrate that SARS-CoV-2 ORF6 determines changes in cellular redox status, leading to a down-regulation of NRF2 and of the NRF2-induced detoxifying system, such as Heme Oxygenase-1 (HO-1) and Glucose-6-Phosphate Dehydrogenase (G6PD) scavengers. As a consequence, the lack of an efficient detoxifying response reported in wild-type (wt)-ORF6 ectopically expressing cells causes intracellular ROS accumulation and compromises the GSH/GSSG ratio. Conversely, the ectopic expression of C-terminal mutated ORF6 variants have minor or no effects. A plausible mechanism of action for ORF6 is issued by RT‒qPCR of messenger RNA for NRF2 and for its downstream scavenger genes that are accumulated following expression of the wt viral protein. We demonstrate that the ORF6 protein hinders host mRNA nuclear export and NRF2 protein translation to establish an unbalanced redox status, which is favourable for virus replication and spread, as described for many other viruses [30, 51]. Indeed, while the expression of the M_58_R and ∆61 protein mutants is negligible for endogenous NRF2 protein accumulation in both HEK-293T and A549 cells, the cytoplasmic protein level of NRF2 is markedly downregulated by wt-ORF6 expression. The antagonistic property of the wt-ORF6 viral protein on NRF2 is further confirmed by investigating the activation of Antioxidant Responsive Elements (ARE)-containing promoter (pARE) present in several antioxidant genes [69, 70]. The wt viral protein targets NRF2-dependent pARE activation, while the loss of activity of the M_58_R and ∆61 ORF6 mutants provides new evidence for SARS-CoV-2 counteracting strategies in host defences. Furthermore, neither the presence of a Keap1 inhibitor (tBHQ), the use of an NRF2 inducer (NAC) nor the overexpression of exogenous NRF2 are able to restore the transcriptional regulation of the ARE promoter when wt-ORF6 and, to a lesser extent, M_58_R is present. Furthermore, the proteasome inhibitor MG-132 fails to restore the levels of exogenous NRF2 when the wt-ORF6 protein is expressed, confirming that the reported decrease in the transcription factor content and downstream activity is not a consequence of its ubiquitin-dependent proteasomal degradation. NRF2 activity can also be regulated at post-translational level through phosphorylation by several kinases. Different kinases are associated with both positive and negative NRF2 regulation, including GSK-3β, PI3K/Akt, MAPKs, β-TrCP and PKC [71–82]. In our hands, wt-ORF6 does not affect PKC and GSK-3β protein levels. Conversely, it induces an increase in phosphorylated, active, p38 MAPK, while the D61 deleted mutant fails to do so, suggesting a role of the C-terminal domain of ORF6 in regulating p38 MAPK activity. Overall, these results provide a new mechanism by which SARS-CoV-2 might modulate cell host homeostasis to favour its own replication and spread, and the present data are strikingly connected to previous observations about IFN-β hindrance by ORF6 [51]. In facts, a recent report demonstrated that NRF2 might be a natural regulator of IFN responses in airway epithelial cells [83] and we speculate that SARS-CoV-2 also takes advantage of the ORF6 protein to minimize the nuclear translocation of residual NRF2 and IFN-β response. This new knowledge supports clinical evidence of a role for oxidative stress in the pathogenesis of COVID-19 and opens the way to new therapeutic strategies, as the viral protein ORF6 represents one of the most important SARS-CoV-2 virulence factors acting as a multifunctional modulator of host cellular processes.

## Conclusions

Oxidative stress has been highlighted in the pathogenesis of SARS-CoV-2 infection and is mainly defined by the overproduction of Reactive Oxygen Species (ROS) which, despite the negative impact on cell metabolism, supports viral replication. Since the mechanisms underlying SARS-CoV-2 pathogenesis are still unclear, we further investigated the activity of the ORF6 protein toward the cellular antioxidant response. Our data demonstrated the antagonistic activity of ORF6 on the transcription factor NRF2, which is associated with the expression of scavenger genes. Thus, ORF6 negatively modulated the cellular detoxifying response and supported the unbalanced cellular redox state, such as the GSH/GSSG redox sensing system. Furthermore, the hindrance of residual NRF2 movement to the nucleus is shown. In conclusion, the NRF2 axis represents a valid target to counteract cell damage induced by a compromised detoxifying system and the exacerbated inflammatory response triggered by the virus.

## Data Availability

Data and materials described in the research will be made available on request.
